# Relationship between chronic kidney disease and sarcopenia

**DOI:** 10.1038/s41598-021-99592-3

**Published:** 2021-10-15

**Authors:** Ming-Dian Yu, Hui-Zhen Zhang, Yu Zhang, Sheng-Ping Yang, Miao Lin, Yan-Min Zhang, Jia-Bin Wu, Fu-Yuan Hong, Wen-Xin Chen

**Affiliations:** 1 Department of Nuclear Medicine, Shengli Clinical Medical College of Fujian Medical University, Fujian Provincial Hospital, No. 134. Dongjie Street, Fuzhou, 350001 China; 2 Department of B-Mode Ultrasound, Shengli Clinical Medical College of Fujian Medical University, Fujian Provincial Jin Shan Hospital, Fuzhou, 350001 China; 3 Department of Nephrology, Shengli Clinical Medical College of Fujian Medical University, Fujian Provincial Hospital, Fuzhou, 350001 China

**Keywords:** Medical research, Nephrology

## Abstract

Few studies have investigated the relationship between sarcopenia and mild to moderate renal decline. This study aimed to investigate the relationship between chronic kidney disease (CKD) and sarcopenia. In total, 123 patients hospitalized with CKD and 57 healthy volunteers who underwent physical examination during the same period (control group) were analyzed. Body compositions were measured by dual-energy X-ray absorptiometry, and the relative appendicular skeletal muscle index (RASMI) was calculated. Muscular strength was evaluated using hydraulic hand dynamometer. Walking speed within 6 m was measured for muscular function assessment. Single-photon emission computed tomography was performed to measure the glomerular filtration rate of CKD patients, who were then divided into CKD1 (55 patients in CKD stages 1 and 2) and CKD2 (68 patients in CKD stages 3–5). RASMI showed a downward trend with CKD progression (*P* = 0.001). Multivariate logistic regression analysis showed that age and CKD progression were independent risk factors for sarcopenia. The morbidity of sarcopenia was significantly greater in CKD patients than in healthy volunteers, and the degree of muscle loss was closely related to CKD progression.

## Introduction

The prevalence of chronic kidney disease (CKD) in the adult population in China is 10.8%; based on this, the number of CKD patients in the existing adult population is estimated to reach 120 million^1^. The incidence of CKD increases with age. With increasing age, humans gradually experience diminished muscle strength accompanied with reduced muscle mass and/or muscle dysfunction, a phenomenon that is often overlooked but extremely common in the elderly; this condition is called sarcopenia. Sarcopenia, a recently discovered geriatric syndrome, is a chronic disease associated with the physiological aging process, where reduced muscle mass, strength, and/or function lead to reduced activity, disability, fall, repeated hospitalizations, and even death in the elderly, seriously affecting the quality of life of the elderly^2^. Although sarcopenia is essentially a disease of advanced age, it may also be secondary to chronic malnutrition, chronic diseases, malignancies, low levels of physical activity, side effects of certain drugs, and so on^3,4^. CKD is one of the major causes and exacerbating factors for sarcopenia^5^. Moreover, the presence of sarcopenia is strongly associated with increased mortality in CKD patients^6,7^. Consequently, full understanding of the relationship between CKD and sarcopenia is of great significance. Although Foley et al.^8^ have verified the correlation between sarcopenia and CKD staging, few researchers have investigated the relationship between sarcopenia and mild to moderate renal decline^9^. Therefore, the present study aimed to analyze the correlations between sarcopenia and patients with early or middle to late CKD.

## Methods

This study was approved by the ethics committee of Fujian Provincial Hospital, all methods were carried out in accordance with relevant guidelines and regulations. This study was carried out in compliance with Declaration of Helsinki. A statement that the study has obtained the informed consent of all participants and/or their legal guardians.

### Study participants

**Figure 1:**
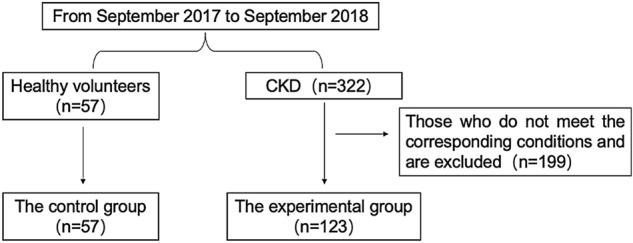
Flow diagram of selection between experimental group and control group.

We conducted a prospective observational study to analyze the correlations between sarcopenia and patients with early or middle to late CKD. A total of 322 patients with CKD who were hospitalized in Fujian Provincial Hospital from September 2017 to September 2018 were selected, among whom patients over 18 years old and non-dialysis dependent chronic kidney disease (CKD-ND) were eligible to participate in the CKD group. The exclusion criteria were as follows: arthritis of both hands or neuromuscular disease, acute disease requiring admission to hospital within the first 6 months, malignancy, grade III or IV congestive heart failure (CHF), severe nephrotic syndrome (defined as weight gain of 5 kg from baseline due to heavy proteinuria), complicated with steroid therapy, Intestinal diseases (such as ulcerative colitis and Crohn’s disease) or other serious organ failure that may affect nutritional status or survival time. The experimental group consisted of 123 CKD patients. 57 healthy volunteers were selected as the control group. The flow diagram is shown in Fig. 1.

Patients were followed up every 1–3 months until December 31, 2019. The end point of this study was death or ESRD (uremia on long-term dialysis). The experimental group and the control group did not withdraw from the study.

### Study methods

All study participants completed the grip strength test, gait speed measurement, and dual-energy X-ray absorptiometry (DXA) examination. Radionuclide renal dynamic imaging was also completed in the CKD group. Glomerular filtration rate (GFR) was obtained by radionuclide renal dynamic imaging, and the CKD group was divided into the following subgroups according to their GFR values; patients with CKD stages 1 and 2 were categorized into the CKD1 subgroup, and those with CKD stages 3–5 were classified into the CKD2 subgroup owing to the small number of cases.

### Data collection

A face-to-face questionnaire survey was conducted on the participants. General data such as name, sex, age (including menopausal age in female), and date of birth; past medical history such as diabetes, hypertension, tumors, and bone diseases; and living habits such as alcohol consumption and smoking were collected.

### GFR measurement

Technetium-labeled diethylenetriamine pentaacetic acid (^99m^Tc-DTPA) with a dose of 185 MBq (5 mCi), which was provided by Fuzhou Branch of Guangdong Shea Pharmaceutical Co., Ltd., was used. Single-photon emission computed tomography (SPECT) (GE Discovery NM/CT 670) unit with low energy high resolution collimator was used to obtain GFR value.

### Laboratory indicators

Fasting blood and urine samples were obtained from each CKD patient within 1 week after enrollment. Serum albumin in plasma was measured using Roche cobas 8000 automatic biochemical analyzer. C-reactive protein (CRP) was measured using Dade Behring BNII specific protein analyzer. The urine dipstick test was performed in all CKD patients.

### Assessment of sarcopenia

#### Walking speed measurement

The method of measuring the walking speed within a 6-m distance was employed. A stopwatch was used to record the time spent walking at a daily pace for 6 m. The walking time was recorded for three times and averaged (t). According to the formula, step speed = distance/time [unit (m/s), the measured step speed was used to evaluate the muscle function^9^.

#### Handgrip strength measurement

The American hydraulic hand dynamometer (JAMAR, Sammons Preston, USA) with a unit of kilogram was used. The measurement was carried out three times with the left and right hands at intervals of more than 1 min to avoid muscle fatigue. Grip strength values were recorded, and the maximum of the six measured values was taken as the grip strength value. Grip strength measurements primarily assess muscle strength^9^.

#### Relative appendicular skeletal muscle index (RASMI) measurement

RASMI was determined by DXA (HOLOGIC, model: Discovery W, USA). In all patients, whole-body scan was performed to obtain patient’s body mass composition, including muscle mass of both upper limbs and lower limbs. The sum of the two is the appendicular skeletal muscle (ASM): RASMI = ASM/height^2^ (kg/m^2^). RASMI is regarded as an evaluation index of muscle mass^9^.

### Definition and diagnosis of sarcopenia

**Figure 2:**
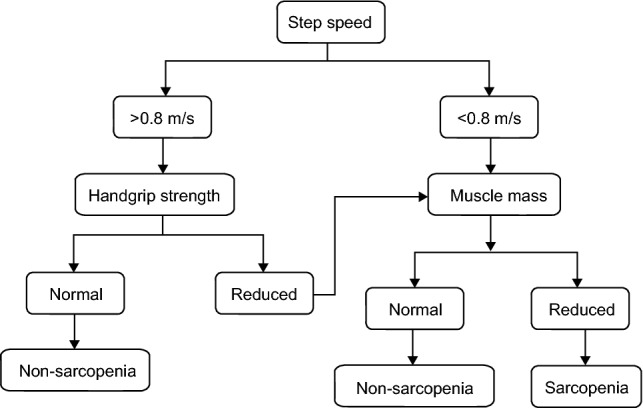
Flow chart of sarcopenia screening and assessment.

According to the diagnostic criteria proposed by the Asian Working Group for Sarcopenia^10^, a skeletal muscle decline combined with low muscle strength and/or muscle dysfunction can be diagnosed as sarcopenia. According to the recommendation suggested by Osteoporosis and Mineral Bone Disease Branch of Chinese Medical Association in 2016^10–13^, the steps for the screening and evaluation of sarcopenia are as follows: (1) First, measure the 6-m step speed; if the step speed is > 0.8 m/s, further test the handgrip strength; if the step speed is ≤ 0.8 m/s, measure the muscle mass in the next step. (2) If the dominant handgrip strength is normal at rest (> 26 kg in male and > 18 kg in female), sarcopenia can be excluded; if the grip strength is less than or equal to the normal values, further measurement of muscle mass is required. (3) If the muscle mass is normal (male > 7.2 kg/m^2^, female > 5.45 kg/m^2^), sarcopenia is excluded; if the muscle mass is lower than the normal value, sarcopenia is diagnosed. The flow chart is shown in Fig. 2.

### CKD stage

According to the Kidney Disease Improving Global Outcomes^14^ guidelines, the CKD stages are defined on the basis of renal impairment, which are as follows: CKD stage 1(G1), GFR ≥ 90 ml/min/1.73 m^2^; CKD stage 2(G2), slightly reduced GFR (60–89 ml/min/1.73 m^2^); In addition, all patients with CKD stage 1 and 2 need to meet dipstick urinary protein scores ≥ 1 + ; CKD stage 3(G3), moderately reduced GFR (30–59 ml/min/1.73 m^2^); CKD stage 4(G4), severely reduced GFR (15–29 ml/min/1.73 m^2^); and CKD stage 5(G5), end-stage renal disease (GFR < 15 ml/min/1.73 m^2^).

### Statistical methods

SPSS 19.0 software (SPSS, Chicago, IL) was used for statistical analysis, and the measurement data were expressed as mean ± standard deviation ($\overline{x} \pm s$). If normal distribution was not met, the Wilcoxon signed-rank test was then adopted. In case of pairwise comparison, the Kruskal–Wallis test was adopted. Enumeration data were expressed as n (%) and compared using the χ^2^ test, and multivariate analysis was performed using logistic regression analysis. *P* < 0.05 was considered statistically significant.

## Results

### General information

In this study, the height, weight and BMI of the experimental group were higher than those of the control group (*P* < 0.001). There was no statistically significant difference in age and sex between the experimental group and the control group, while the 6-m step speed, grip strength, RASMI and total GFR in the experimental group were significantly lower than those in the control group (all *P* < 0.05). The morbidity of sarcopenia was significantly greater in CKD patients than in healthy volunteers (*P* < 0.001), as shown in Table 1.

**Table 1:** Comparison of the general characteristics between the experimental group and control group.

	Experimental group (n = 123)	Control group (n = 57)	*P*
Age (years)	59.31	58.16	0.56
Sex (M/F)	61/62	26/31	0.63
Height (cm)	162.0	158.8	0.01
Weight (kg)	62.98	56.23	< 0.001
BMI (kg/m^2^)	23.89	22.17	< 0.001
Step rate (m/s)	0.84	0.99	< 0.001
Grip strength (kg)	48.28	80.46	0.001
RASMI (kg/m^2^)	5.30	6.22	< 0.001
Sarcopenia (n)	60	11	< 0.001

*BMI* body mass index, *RASMI* relative appendicular skeletal muscle index.

The age of CKD patients in the sarcopenic group was higher than that of the non-sarcopenic group (*P* < 0.001), and the incidence of sarcopenia was 55.7% in male and 41.9% in female in the CKD group, with no significant difference. No significant differences were found in height, weight, and body mass index (BMI) between the sarcopenic and non-sarcopenic groups, whereas the 6-m step speed, grip strength, RASMI, and total GFR were significantly lower in the sarcopenic group than in the non-sarcopenic group. The muscle mass of both upper and lower limbs and ASM were significantly lower in the sarcopenic group than in the non-sarcopenic group (all *P* < 0.05). The smoking rate, positive of urinary protein, concurrent diabetes rate and hypertension rate in sarcopenia group were higher than those in non-sarcopenia group (all *P* < 0.05), as shown in Table 2.

**Table 2:** Comparison of the general characteristics between the sarcopenic and non-sarcopenic patients in the CKD population.

	Sarcopenia (n = 60)	Non-sarcopenia (n = 63)	*P*
Age (years)	64.25 ± 11.32	54.60 ± 14.25	< 0.001
Sex (M/F)	34/26	27/36	0.162
Height (cm)	161.6 ± 8.55	162.35 ± 7.79	0.595
Weight (kg)	62.35 ± 12.40	63.58 ± 9.25	0.340
BMI (kg/m^2^)	23.64 ± 3.99	24.06 ± 2.72	0.180
Step rate (m/s)	0.72 ± 0.24	0.94 ± 0.13	< 0.001
Grip strength (kg)	18.48 ± 8.99	26.06 ± 7.51	< 0.001
RASMI (kg/m^2^)	5.03 ± 0.86	5.55 ± 0.72	< 0.001
Total GFR (ml/min)	42.81 ± 32.00	56.96 ± 30.62	0.014
Muscle mass of both upper limbs (g)	3737.84 ± 970.53	4070.47 ± 990.55	0.048
Muscle mass of both lower limbs (g)	9485.36 ± 2268.07	10,639.44 ± 1930.54	0.002
ASM (g)	13,223.20 ± 3037.12	14,709.91 ± 2626.61	0.004
Urine protein (+)	39	24	0.03
ALB2 (g/l)	37.0	38.0	0.43
CRP (mg/l)	27.28	30.28	0.82
Fasting blood glucose (mg/dl)	7.20	6.74	0.39
Current smoker (%)	75%	58.7%	0.06
Current drinking (%)	60%	46%	0.15
Medical history (n, %)			
Chronic glomerulonephritis (%)	25.5%	37.8%	0.26
Interstitial nephritis (%)	7.7%	14.6%	0.48
DM (%)	46.7%	27.0%	0.03
Hypertension (%)	75.0%	33.9%	< 0.001
Polycystic kidney (%)	16.3%	22.6%	0.46

*ASM* appendicular skeletal muscle, *BMI* body mass index, *RASMI* relative appendicular skeletal muscle index, *total GFR* glomerular filtration rate, *ALB2* Serum albumin; *CRP* C-reactive protein, *DM* diabetes.

### Logistic regression analysis of related factors of sarcopenia

With sarcopenia (yes = 1, no = 0) as the dependent variable. with age, CKD (G1 = 1, G2 = 2, G3 = 3, G4 = 4, G5 = 5), urinary albumin, serum albumin and hypertension (yes = 1, no = 0) as independent variables, Multivariate step-by-step logistic regression analysis (Wald Backward) showed that age and CKD were independently correlated with sarcopenia.: for every 1-year increase in age, the risk of sarcopenia increased by 6.9% (OR = 1.069, *P* < 0.001), and for each additional grade of CKD, the risk of sarcopenia increased by 45% (OR = 1.45, *P* = 0.013), as shown in Table 3.

**Table 3:** Logistic regression analysis of related factors of sarcopenia.

Independent variable	OR value	95% C.I. for OR		*P* value
		Lower	Upper	
Age	1.069	1.032	1.107	< 0.001
CKD	1.450	1.080	1.945	0.013

*CKD* chronic kidney disease stages 1–5, *CI* confidence interval, *OR* odds ratio, *SE* standard error.

### Analysis of the relationship between GFR and RASMI

GFR was measured by radionuclide renal dynamic imaging, and CKD patients were divided into the CKD1 and CKD2 groups according to GFR. RASMI was significantly decreased with the progression of CKD by unpaired t-test, and the difference was statistically significant (Z =  − 3.253, *P* = 0.001), as shown in Table 4.

**Table 4:** Analysis of the relationship between GFR and RASMI.

Renal function tests	Subgroup	Number of cases	RASMI (kg/m^2^)	Z	*P*
GFR	CKD1 subgroup	55	5.59 ± 0.79	− 3.253	0.001
	CKD2 subgroup	68	5.06 ± 0.80		

*CKD* chronic kidney disease, *GFR* glomerular filtration rate; *RASMI* relative appendicular skeletal muscle index.

## Discussion

CKD is a growing global health problem and is a catabolic state known to be associated with protein consumption and various metabolic disorders due to uremia^15^, resulting in reduced skeletal muscle anabolism and increased catabolism, making CKD patients more susceptible to sarcopenia.

Sarcopenia can be divided into primary and secondary sarcopenia. Primary sarcopenia is associated with aging and is a process of physiological aging, but its process is affected by lifestyle, environmental factors, genetic factors, and so on, with significant individual differences^16^. Secondary sarcopenia can be divided into disease-related, nutrition-related, and activity-related sarcopenia. As CKD progresses, CKD patients may have secondary decreased protein intake, metabolic acidosis, increased pro-inflammatory factors, decreased growth hormone and sex hormones, and protein-energy wasting (PEW), and may lack physical activity, myostatin overexpression, and decreased insulin and insulin-like growth factor levels. All these factors change to varying degrees from the early-stage CKD to the dialysis stage, with reduced skeletal muscle strength, skeletal muscle fiber mass, and lower muscle endurance and metabolic capacity, namely, CKD sarcopenia^17,18^. CKD-associated sarcopenia is caused by an altered balance of skeletal muscle catabolism and anabolism on controlling muscle homeostasis, which is a very complex process^19^. In this study, we divided CKD into stages 1–5, and logistic regression analysis showed that CKD stage was independently associated with sarcopenia, and the risk of sarcopenia increased by 45% for each grade of CKD progression, showing that CKD is a very important risk factor causing sarcopenia.

Foreign studies on the relationship between CKD and sarcopenia have been carried out in recent years, but most of them focused on participants with end-stage renal disease (ESRD) undergoing hemodialysis. Domanski et al.^20^ suggested that in CKD patients, the reduction of muscle mass was more severe and earlier than that in their peers, in addition to the fact that sarcopenia was more common in patients with end-stage renal disease. Kim et al.^21^ defined sarcopenia according to the criteria of the European Working Group on Sarcopenia in Older People, and the results of their study showed that the prevalence of sarcopenia was quite common in elderly patients with end-stage renal disease. This study found that the prevalence of sarcopenia is high not only in patients with advanced CKD, but in patients with early stages of CKD; thus, attention should be paid to the incidence of sarcopenia in patients with early stage of CKD.

In addition, the incidence of sarcopenia was higher in male than in female CKD patients, consistent with the findings of Lamarca et al.^22^. This might be related to sex hormones, as androgen (testosterone) maintains muscle mass by mediating protein synthesis. Interstitial cells secrete testosterone, which may affect the formation/regeneration of skeletal muscle, and studies have demonstrated that testosterone increases the number of satellite cells and stimulates muscle protein synthesis^23^. In CKD patients, male hypogonadism is common and may be exacerbated by other common CKD comorbidities (i.e., obesity, diabetes, and hypertension)^24^. Testosterone levels are associated with reduced muscle mass and strength in CKD^18,25^, and researchers have found that nandrolone decanoate is associated with improved skeletal muscle mass in randomized controlled trials in dialysis patients^26^. Maric et al.^27^ found that testosterone levels were significantly decreased, and estradiol levels were increased in male patients with diabetic nephropathy. Changes in hormonal levels in the body increase the susceptibility of male CKD patients to sarcopenia. In this study, although the incidence of sarcopenia in men was significantly higher than that in women, the difference was not statistically significant, which was possibly due to the small sample size.

GFR is an important factor in CKD staging, and Zhou et al.^28^ found that for every unit decrease in GFR, muscle mass was reduced by 0.15 ± 0.07 kg and RASMI by 0.03 ± 0.01 kg/m^2^. In this study, RASMI decreased significantly with the progression of CKD, which is consistent with that reported by Zhou et al., but the difference is that Zhou measured GFR by iohexol, whereas the present study adopted renal dynamic imaging using ^99m^Tc-DTPA^29^. SPECT ^99m^Tc-DTPA renal dynamic imaging cannot only accurately measure GFR, but can also obtain relevant information such as renal function, excretion, shape and size of both kidneys, and the presence or absence of obstruction.

PEW^30^ is common in CKD patients, especially in end-stage dialysis patients, and most patients have protein-energy undernutrition, which can present as a syndrome characterized by a micro inflammatory state, low BMI, progressive skeletal muscle wasting, and inadequate nutritional and caloric intake. The skeletal muscle is the largest organ in the body that stores protein and can be regarded as an important indicator of protein and energy deficits in CKD patients. CKD patients often exhibit loss of appetite, and some patients even suffer from anorexia. Moreover, patients’ daily intake of food is reduced, which leads to undernutrition and reduced plasma albumin levels, thereby affecting the synthesis and metabolism of muscle proteins. A low protein diet has long been recommended for CKD patients, so that patients have a serious lack of protein intake, affecting the synthesis of protein in the body, resulting in reduced muscle mass, which in turn causes decreased muscle strength. In this study, the serum albumin level of sarcopenia patients was lower than that of non-sarcopenia patients, but the difference was not statistically significant, which may be related to the small sample size included.

Currently, the most accurate test for proteinuria is timed (usually 24 h) urine collection for quantification. However, this method is not only time-consuming, it can also be imprecise. Therefore, current clinical practice guidelines recommend urinary creatinine-adjusted “spot urine total protein or albumin” as the best method for evaluating proteinuria, which is also time-consuming and expensive. Urine dipstick test, by contrast, because of its low cost, wide availability, the result rapidly and is widely used as proteinuria detection of initial screening tool^31^. Lim et al. to explore the accuracy of urine dipstick in detecting proteinuria in patients. They concluded that if the albumin/creatinine ratio (ACR) ≥ 30 mg/g or the protein/creatinine ratio (PCR) is intended as a reference criterion for proteinuria, the urine dipstick test may be recommended for screening. Although there are no ACR and PCR reference criteria for urinary protein in this study to be compared with the urine dipstick test, the urine protein dipstick test is an adequate measure of urinary protein^31^. Therefore, we screened CKD1 and 2 patients by dipstick urinary protein scores ≥ 1 +^32^.Previous studies have shown that proteinuria is more common in muscular dystrophy patients than in non-muscular dystrophy patients, regardless of hypertension, diabetes, or obesity^33^. Our study found similar results in univariate analysis in patients with CKD.

The strengths of this study include the adoption of DXA to determine the appendicular skeletal muscle mass (ASM) of the four limbs. Subsequently, RASMI^34^ is derived by dividing the ASM by the square of height, which is then used as an important indicator for the diagnosis of sarcopenia. Since the ASM of the four limbs are not only the most relevant functional part of lean body weight, but also remain uninfluenced by variations in the lean body weights of organs, changes in the patient’s muscle mass can be more accurately reflected. In addition, compared with other examination devices, DXA is easier to use and provide more accurate results. Another advantage of the study is that the GFR measured by ^99m^Tc-DTPA renal dynamic imaging is not only a more appropriate indicator of the renal function of CKD patients, but also capable of monitoring the patient’s differential renal function.

However, this study had some deficiencies. First, Due to a small sample size, CKD patients were only categorized into an early-stage group and a middle- to late-state group for analysis. Second, as questionnaires were used to collect relevant patient information, the possibility of information bias cannot be excluded. Third, the accuracy of this study’s determination of proteinuria may be doubtful because it defined using a one-time urine dipstick test. Sensitivity to low or higher albuminuria depends on such factors as standard ACR ratios and automatic reading measurement criteria^31^. To overcome this, dipstick screening must be repeated; ACR urine marker proteinuria standard should also be developed and accurate reading of the measurement standard. Finally, the criteria for our study to diagnose sarcopenia was AWGS2014, not AWGS2019, although AWGS2019 is currently under implementation period.

In summary, CKD patients are more susceptible to sarcopenia, which is common in all stages of CKD. These may easily cause fracture, fall, disability, hospitalization, and increased cardiovascular morbidity in CKD patients, and even lead to death, imposing a large burden to the individual and society. Therefore, it is of great social significance to diagnose CKD with sarcopenia as early as possible. At this time, the development of corresponding treatment measures may be able to reverse the process of muscle loss, thereby preventing the complications of CKD due to sarcopenia and improving the quality of life of patients, which is of great social significance.

## Data Availability

The datasets used and analysed during the current study are available from the corresponding author on reasonable request.
